# Childhood trauma, psychotic symptoms: which association?

**DOI:** 10.1192/j.eurpsy.2023.2205

**Published:** 2023-07-19

**Authors:** A. Syrine, F. Rim, G. Imen, S. Najeh, O. Sana, M. B. Manel, Z. Lobna, B. T. Jihen, C. Nada, M. Mohamed

**Affiliations:** Psychiatry ‘C’ Department, University Hospital of Hedi Chaker, Sfax, Tunisia

## Abstract

**Introduction:**

Clinical evidence supports the interaction between genetic predisposition and environmental stressors on the emergence of positive psychotic symptoms. Childhood trauma might be a modifiable risk factor among adults with serious mental illness.

**Objectives:**

The aim of our study was to investigate associations between childhood trauma (physical abuse, sexual abuse, emotional abuse, emotional neglect, and physical neglect) and symptoms of schizophrenia.

**Methods:**

We included in our study 33 stabilized inpatients with schizophrenia at the Psychiatry C department at University Hospital in Sfax-Tunisia.

Data on Sociodemographic and clinical variables were collected from medical records.

Psychotic symptoms were evaluated using the Positive and Negative Syndrome Scale (PANSS). We used the Childhood Trauma Questionnaire-Short Form (CTQ-SF) to evaluate childhood trauma experiences.

**Results:**

Our sample was exclusively composed of men with an average age of 35 years and 4 months.

The majority of patients were unemployed (75.8%). Only 6.1% of them were married.

Among the patients included in the study, 15.2% of our patients were illiterate. The majority of subjects were treated after a period of untreated psychosis (65.5%).

The analysis revealed that 42.4% of our patients experienced childhood adversities with mean CTQ-SF total score 35.48 (SD=9.44)

We found that positive and general psychopathology schizophrenia symptomatology were correlated to Childhood abuse (0.03; 0.004), emotional abuse (0.009; 0.004), physical neglect (0.02; 0.01), and emotional neglect (0.01).

In addition, our analysis showed that only emotional abuse lead to more negative schizophrenia symptomatology (p=0.009).*

**Conclusions:**

Several studies have shown an association between childhood trauma and specific symptoms of psychosis. Therefore, Childhood trauma should be considered and inquired about in the course of clinical care of schizophrenia patients.

**Disclosure of Interest:**

None Declared

